# Bibliometric analysis of the usage of tenecteplase for stroke

**DOI:** 10.1186/s12245-024-00738-7

**Published:** 2024-11-01

**Authors:** Garv Bhasin, Latha Ganti

**Affiliations:** 1 Brown University, Providence, RI USA; 2 Orlando College of Osteopathic Medicine, Winter, FL 34787 USA; 3 Warren Alpert Medical School of Brown University, Providence, RI 02903 USA

**Keywords:** Tenecteplase, Acute ischemic stroke, Bibliometric analysis

## Abstract

**Introduction:**

In recent years, tenecteplase has been competing with alteplase as a treatment for acute ischemic stroke given its ease of administration, lower dosage, cost-effectiveness, and better safety data. This paper seeks to analyze academic literature regarding the burgeoning usage of tenecteplase as a treatment for acute ischemic stroke across the world.

**Method:**

The Web of Science database was used to collect the data from articles containing the keywords “Tenecteplase” and “Stroke” published from 1999 to 2023. The search resulted in 576 journal articles. This study analyzed metadata related to the country, institution, keywords, and date published for each article in the database pertaining to tenecteplase use for stroke.

**Results:**

The United States led in publications (260, 39.93%), followed by Australia (101, 15.51%), and a tie for third place between Canada and China (77, 11.83% each). The three most prevalent keywords were tenecteplase (*N* = 324), alteplase (*N* = 284), and thrombolysis (*N* = 244). The University of Melbourne and the University of Calgary were the leading institutions publishing on the use of tenecteplase as a treatment for stroke. In 2023, the number of publications on the usage of tenecteplase for stroke was the greatest, making up 24.3% of all papers on the topic.

**Conclusion:**

The surge in academic papers regarding tenecteplase in stroke in 2023 could be a good indicator of the drug’s increasing prevalence as a treatment for stroke. Despite this finding, tenecteplase is currently not an FDA-approved therapy in the US as Genentech, the drug’s manufacturer, has yet to file for federal approval for acute ischemic stroke treatment.

## Introduction

Worldwide, strokes are the third leading cause of disability and the second leading cause of death [1]. Strokes alone are responsible for people losing 143 million disability-adjusted life years worldwide [2]. Disability-adjusted life years are a measurement of the overall burden of a disease and are calculated by combining the years of life lost due to premature mortality or healthy life lost due to disability [3]. Due to aging populations, the burden of stroke has increased rapidly, particularly in low-income populations around the world [1]. From 2011 to 2021, the United States saw a 26.3% increase in stroke-related deaths [4]. Stroke may occur in two different ways: acute ischemic strokes and hemorrhagic strokes. This paper will focus on acute ischemic strokes and the recent increase in administering tenecteplase to treat acute ischemic strokes as opposed to utilizing its predecessor, alteplase.

Tenecteplase is a tissue Plasminogen Activator (tPA) most widely used to treat ST-segment-elevation myocardial infarctions (STEMIs) [5]. Tenecteplase has also been used as an off-label treatment for acute ischemic strokes. In recent years, tenecteplase has outperformed alteplase, the only FDA approved thrombolytic used for acute ischemic stroke, primarily due to its better safety data, cost-effectiveness, the lower dosage required to treat strokes, and ease of administration [6, 7]. A study comparing the cost-effectiveness of alteplase and tenecteplase in the Dutch healthcare system found that over 10 years, tenecteplase was associated with gaining 0.05 Quality-adjusted life years while saving €21 per patient [8]. Quality-adjusted life years measure how well medical treatments lengthen or improve patients’ lives [9]. Another study performed a meta-analysis to evaluate the safety and efficacy of tenecteplase versus alteplase and found no difference between the two drugs in early neurological improvement, neurological recovery, and mortality supporting the finding that tenecteplase is non-inferior to alteplase in terms of safety outcomes [10]. Despite its efficacy in stroke treatment, tenecteplase is not yet an FDA-approved treatment for acute ischemic stroke and is only used as an off-label treatment. This paper seeks to underscore the rising importance of tenecteplase in treating acute ischemic strokes in patients. Furthermore, analyzing the prevalence of tenecteplase is important given the extensive research currently underway across the United States that has the potential to initiate a formal institutional shift from using alteplase to tenecteplase as the primary treatment for acute ischemic stroke [11].

## Methods

The Web of Science database was used to perform the analysis as it is an established database containing data on a wide array of topics, covering publications dating back to 1990 [12]. The keywords Tenecteplase and Stroke (TS = “Tenecteplase” AND “Stroke”) were used to perform the search. Data was collected from 1999 to 2023, excluding 2024 since at the time of writing this paper there was an incomplete amount of data for the year 2024. The search resulted in 576 journal articles. The data was exported as tab-delimited files from Web of Science to a desktop application called VosViewer, a tool used for the analysis of bibliometric data. This study utilized Vosviewer version 1.6.20 to assist in the creation of various graphs to better visualize the data. Graphs were created for the number of publications produced by country, the number of occurrences for keywords, the number of publications produced by institutions across the world, and the most prolific articles on tenecteplase usage in stroke. To prevent overcrowded graphs, the minimum number of papers published for countries to be included in the graph was seven, the minimum number of occurrences for a keyword to be included in the graph was 45, the minimum number of citations for a document to be included in the analysis was 100, and the minimum number of occurrences for an institution to be included in the graph was 16.

## Results

### Countries and regions

**Fig. 1:**
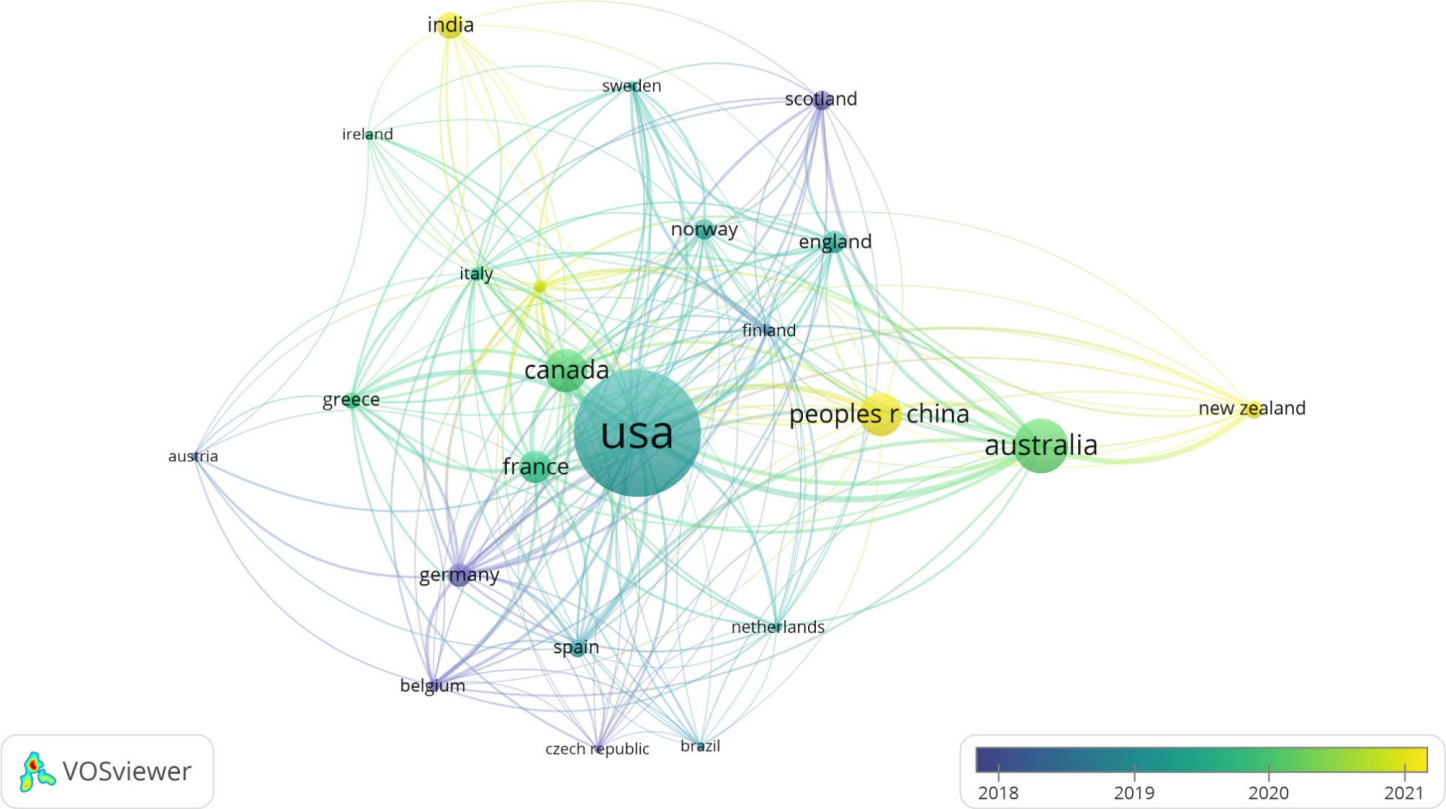
Publication data by country for tenecteplase for stroke

The United States (US) was the country with the greatest number of publications having published 260 articles on tenecteplase in stroke and accounting for about 39.93% of articles published on the topic. The US was followed by Australia which published 101 (15.51%) of the papers on tenecteplase in stroke. China and Canada were tied for the third greatest number of publications on the topic with 77 publications each (11.83%). As shown in Fig. 1, countries that were closer together were more likely to collaborate on co-authorship in papers. According to Fig. 1, even though the US had the greatest number of publications, the majority of the publications were comparatively older having been published in 2019 or 2020. Countries with fewer but more recent publications on tenecteplase usage in stroke included India, the People’s Republic of China, and New Zealand indicating a recent interest in tenecteplase usage to treat stroke in those countries.

**Fig. 2:**
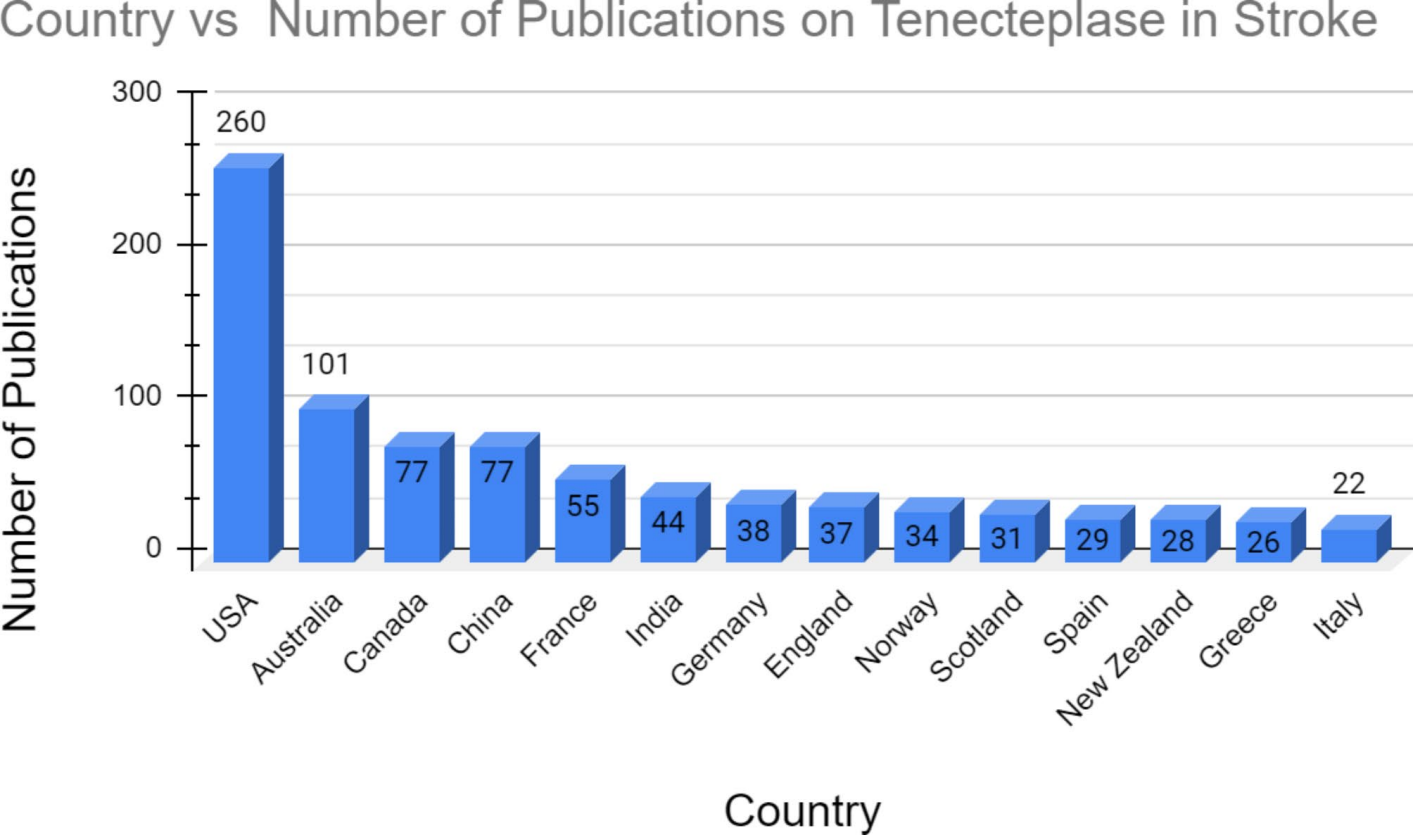
Bar graph for publication data by country for tenecteplase in stroke

An important point to note when looking at Fig. 2 is that adding all the publications produced by each country will not result in a total of 576 publications. This is because the collaboration of individuals and institutions across country lines on certain publications would mean that the Web of Science would catalog those publications as belonging to multiple countries. The discrepancy in numbers can be attributed to this mechanism of redundant categorization.

### Keyword usage

**Fig. 3:**
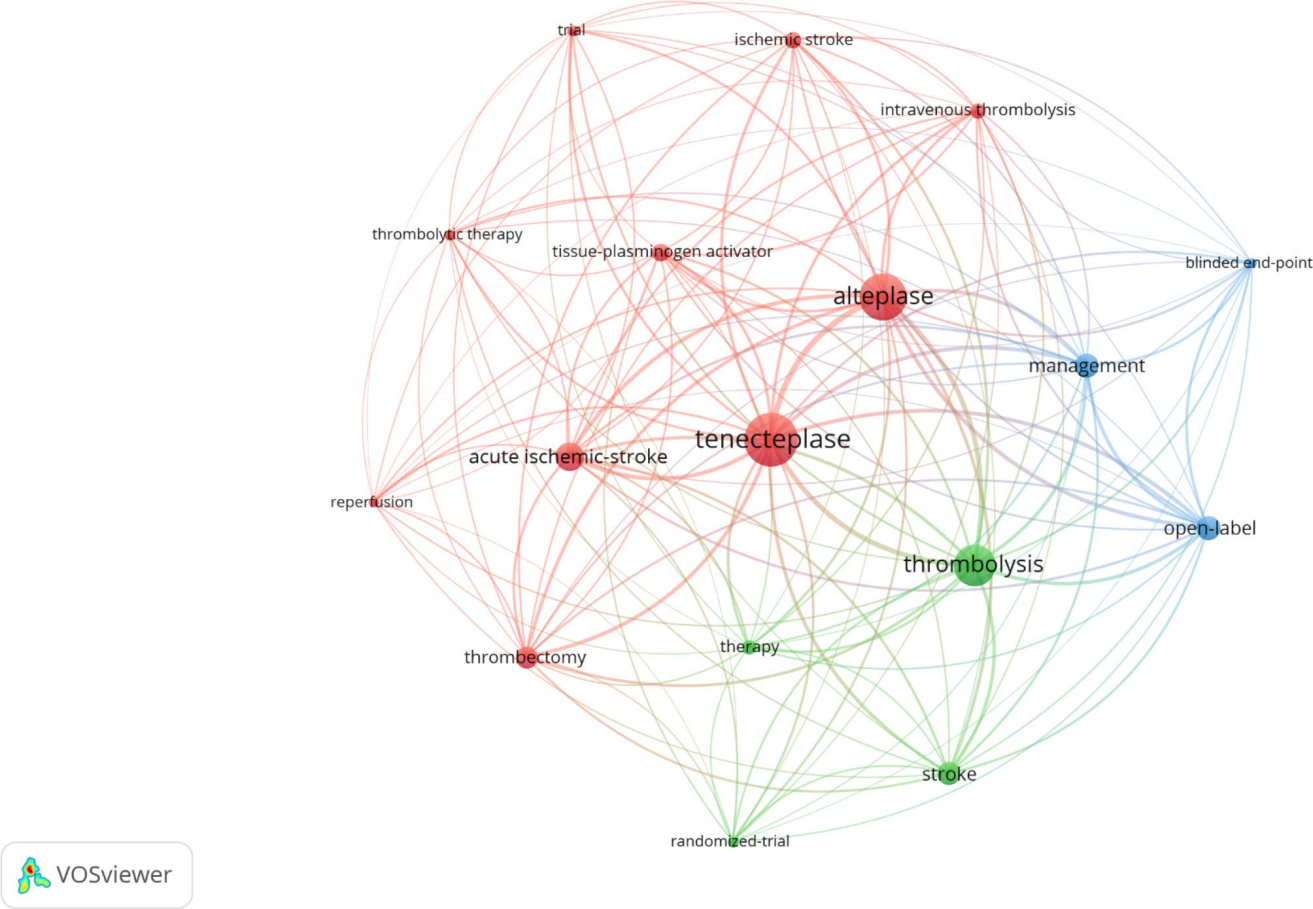
Publication data by keyword for tenecteplase for stroke

The keywords [Fig. 3] that showed up most often in the papers were tenecteplase (*N* = 324), alteplase (*N* = 284), thrombolysis (*N* = 244), and acute ischemic-stroke (*N* = 161).

### Institutions and organizations

**Fig. 4:**
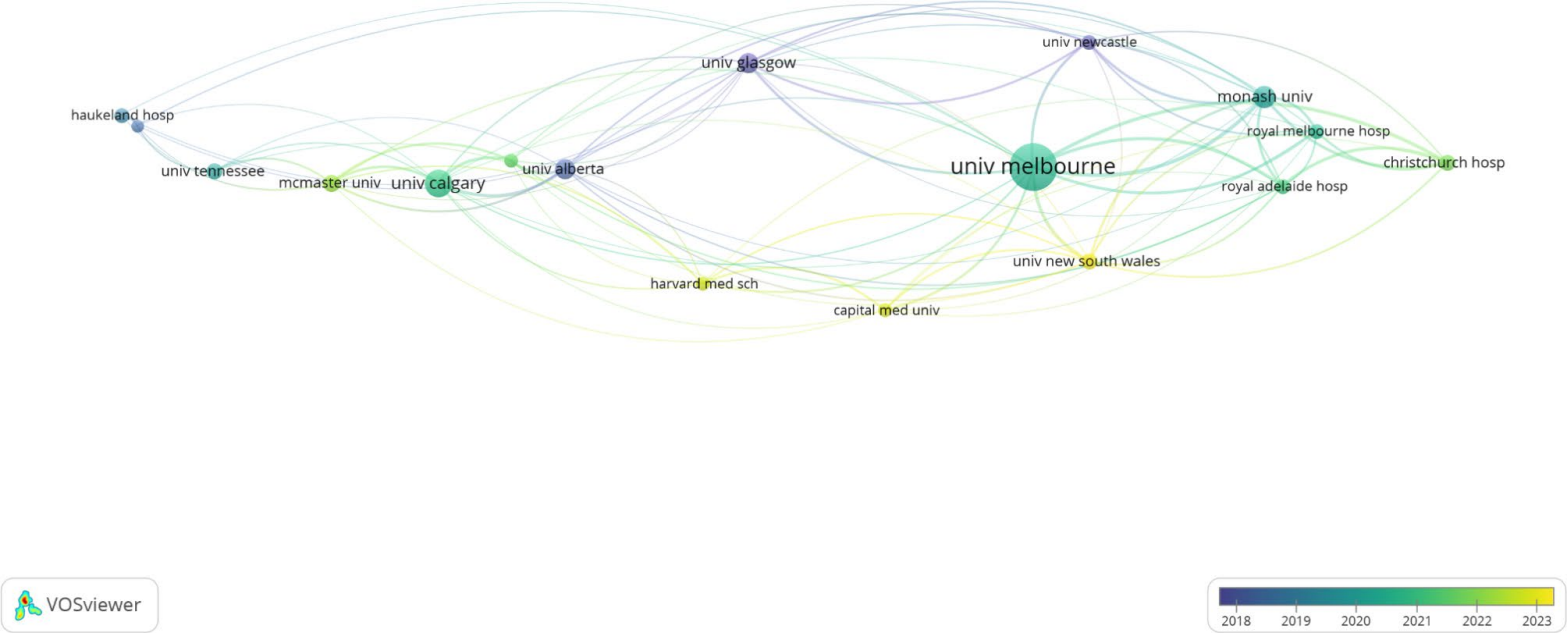
Publication data for Institutions that have published on Tenecteplase for Stroke

**Fig. 5:**
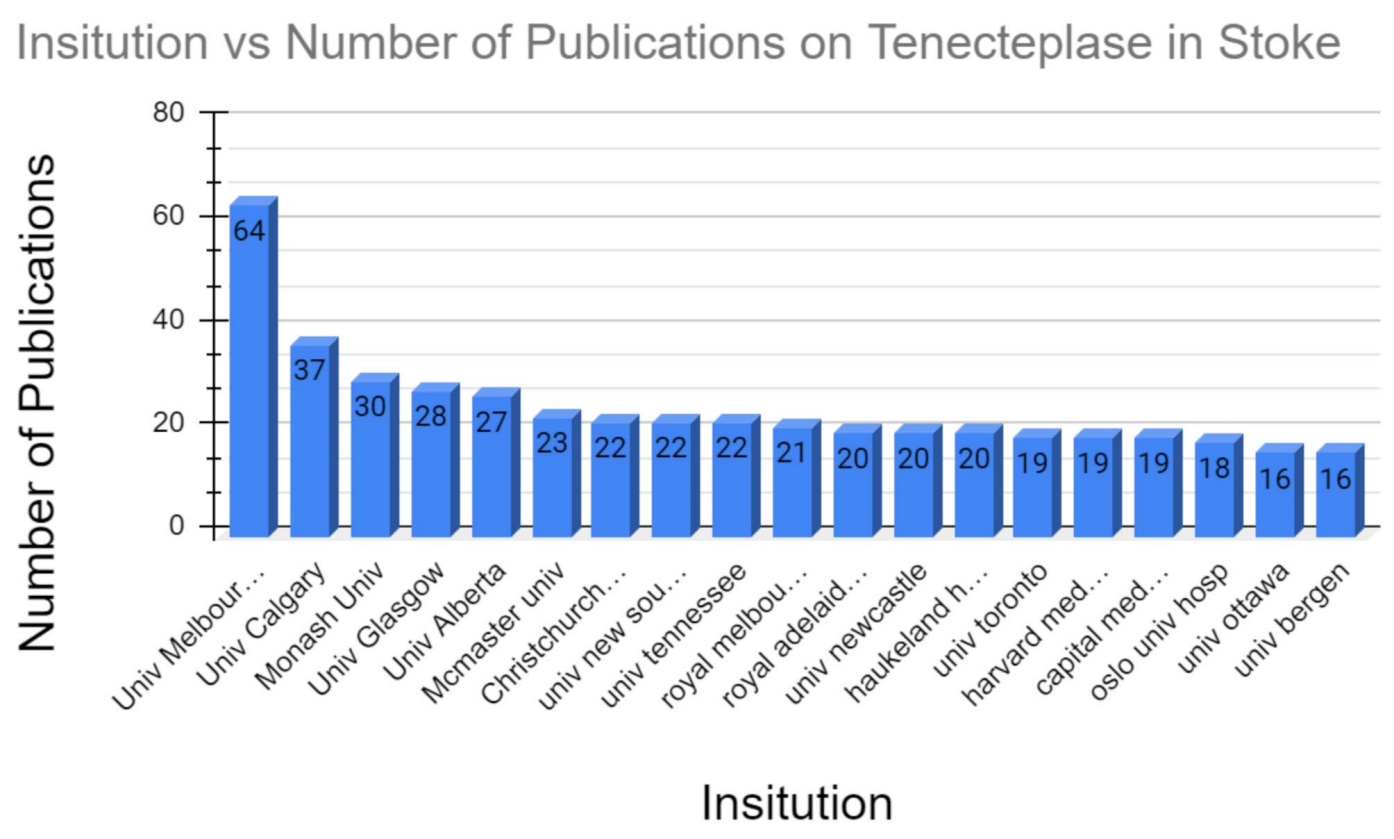
Bar graph data for institutions that have published on tenecteplase for stroke

Several prolific institutions across the world have published papers on tenecteplase usage in stroke [Fig. 4]. The institution with the greatest number of publications on tenecteplase in stroke was the University of Melbourne with 64 publications. The second and third institutions with the greatest number of publications on the topic were the University of Calgary and Monash University with 37 and 30 publications respectively. The University of Glasgow came in fourth place with 28 publications. An important note in this section was that the trend in the distribution of publications produced by institutions follows a similar pattern to the distribution of publications produced by countries. In both the country and institution datasets (Figs. 2 and 5), there are outliers that have published significantly more papers on tenecteplase usage in stroke relative to the rest of the data set (i.e. the USA in the country dataset and the University of Melbourne in the institution dataset). However, after the top 3 publishers in both fields, publications are relatively evenly spread among the remaining countries and institutions.

### Yearly data

**Fig. 6:**
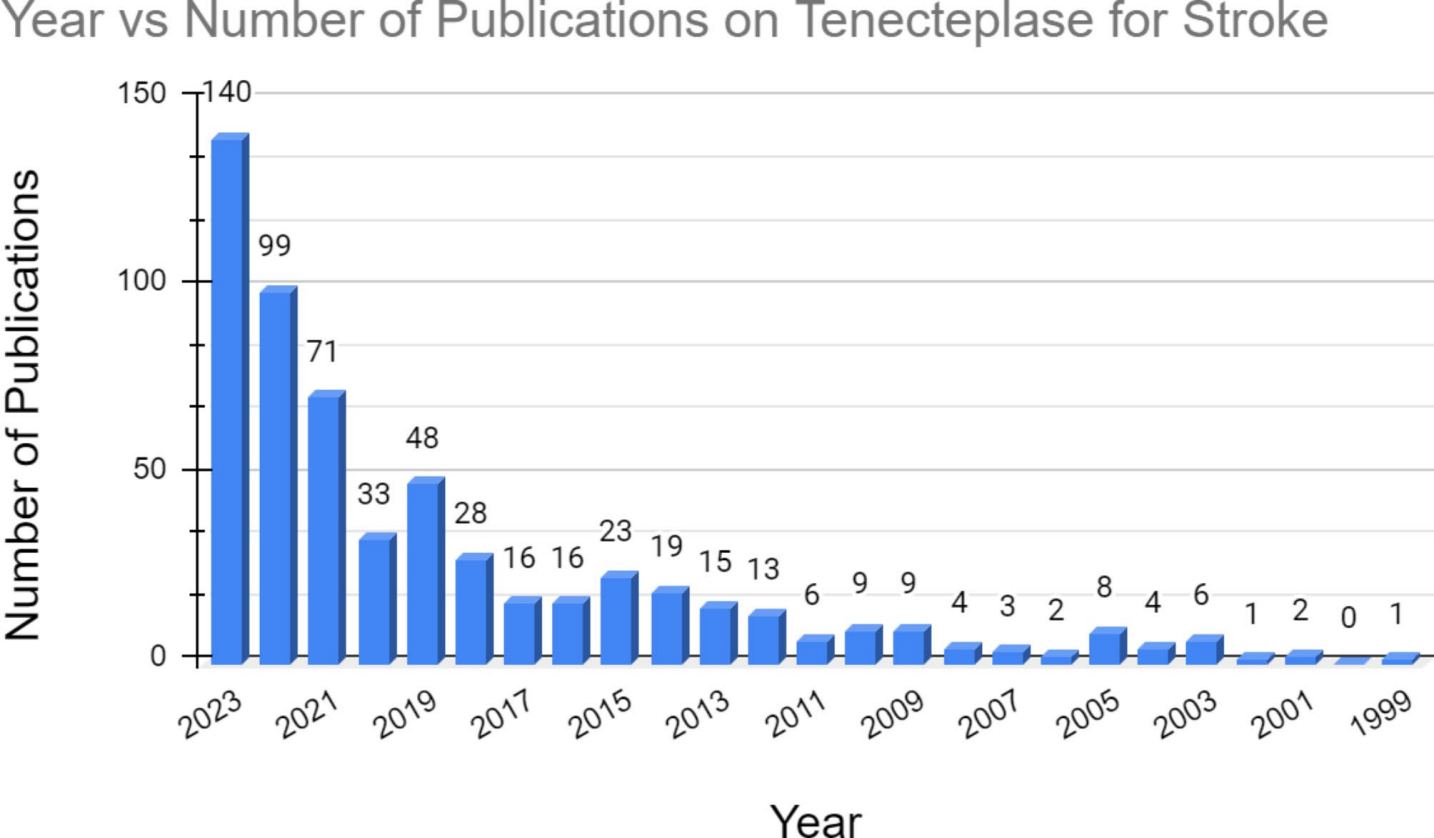
Bar graph showing the yearly trend in publications on tenecteplase in stroke

The year 2023 had the greatest number of publications on tenecteplase in stroke, accounting for 24.3% (*N* = 140) of all publications on the topic [Fig. 6]. The greatest increase in the number of publications on tenecteplase in stroke occurred between the years 2022 (*N* = 99) and 2023 (*N* = 140). Figure 5 shows that there has been a steady increase in the number of publications on tenecteplase in stroke from 2012 to 2023, indicating a growing interest in the topic.

### Tree map categories for teneceteplase in stroke

**Fig. 7:**
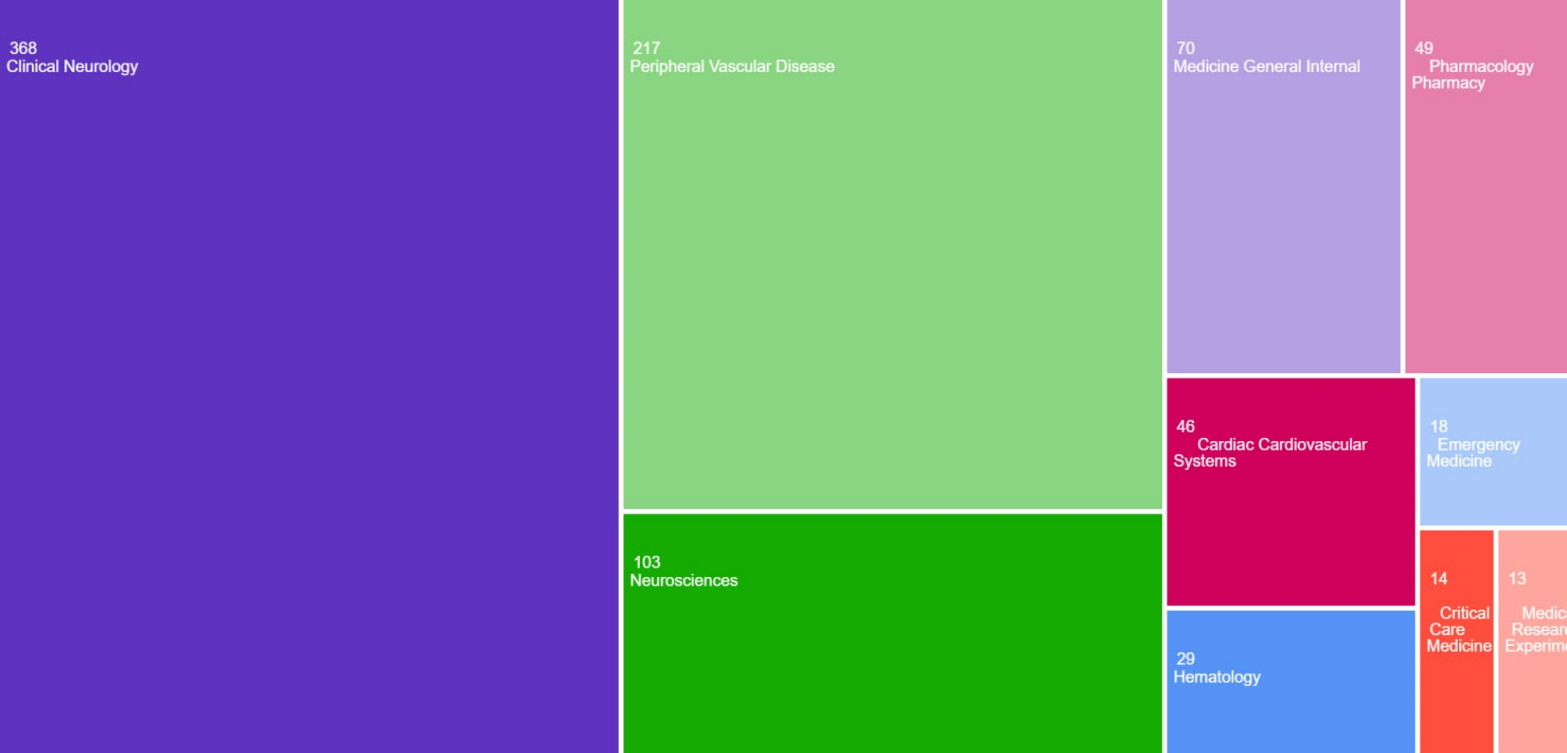
Tree map chart categorizing publications on tenecteplase in stroke

Web of Science visualizes the categories associated with a certain topic using a tree map. The articles were mainly categorized under the fields of Clinical Neurology (39.7%), peripheral vascular disease (23.4%), Neuroscience (11.1%), Medicine General Internal (7.56%), and Pharmacology Pharmacy (5.29%) [Fig. 7]. All other categories only accounted for 12.9% of the article categorizations for tenecteplase usage in stroke.

### Prolific articles

**Fig. 8:**
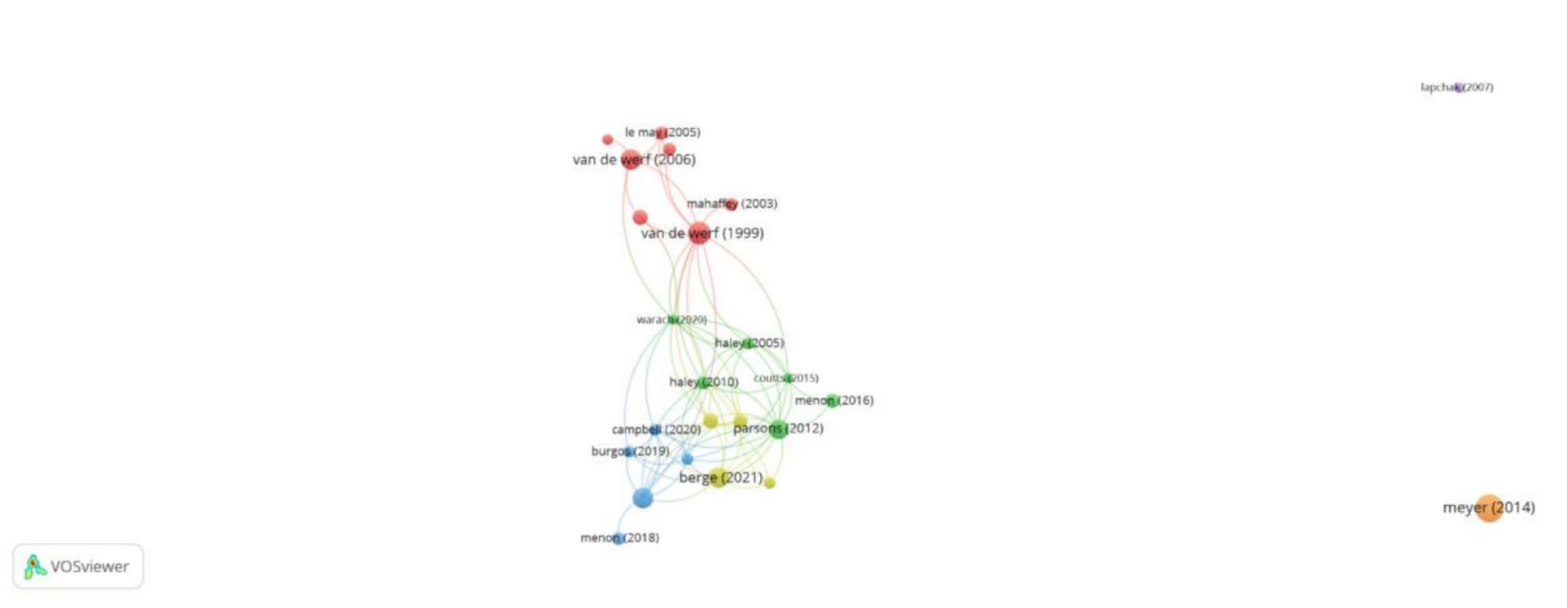
Graph showing prolific articles published on tenecteplase in stroke

The article with the most citations regarding tenecteplase in stroke was “Fibrinolysis for Patients with Intermediate-Risk Pulmonary Embolism” (998 citations) by Meyer et al. published in 2014 [13] [Fig. 8]. The article with the second most citations was “Single-bolus tenecteplase compared with front-loaded alteplase in acute myocardial infarction: the ASSENT-2 double-blind randomised trial” (624 citations) by Van de Werf et al. published in 1999 [14]. The article with the third most citations was “European Stroke Organisation (ESO) guidelines on intravenous thrombolysis for acute ischaemic stroke” (503 citations) by Berge et al. published in 2021 [15]. The article with the fourth most citations was “Primary versus tenecteplase-facilitated percutaneous coronary intervention in patients with ST-segment elevation acute myocardial infarction (ASSENT-4 PCI): randomised trial” (500 citations) by Van de Werf et al. published in 2006 [16]. The article with the fifth most citations was “Tenecteplase versus Alteplase before Thrombectomy for Ischemic Stroke” (487 citations) by Campell et al. published in 2018 [17].

## Discussion

### Countries

A longitudinal study in the US found that thrombolysis as a treatment for stroke has increased from 10 to 15% in 2003 to 43 to 46% in 2021 across all racial and ethnic groups present in the sample [18]. The usage of thrombolytic agents to treat stroke in the US healthcare system has increased since 2003, and as such, the research on major thrombolytic agents such as teneceteplase to treat stroke would have also increased. This data could explain why the US is the leading country in publications on tenecteplase usage in stroke. Australia is the country with the second most publications on tenecteplase usage in stroke. Stroke research has the potential to significantly improve healthcare outcomes in Australia, where stroke is the third leading cause of death [19]. From 2007 to 2011, Australia increased the number of stroke units offering thrombolysis from 24 to 36% [20]. Furthermore, in the year 2021, Australia spent about 984 million Australian dollars on strokes [21]. The large amounts of money the Australian government spends on stroke likely enable it to be a leader in publications on tenecteplase usage in stroke. The USA, Australia, and Canada are all large countries with a considerable population living in rural areas. As such, these countries could have an incentive to perform studies on tenecteplase as it would improve stroke outcomes in rural areas due to tenecteplases’ administration over a shorter time (5-second single bolus as opposed to alteplase which is a dose administered over 60-minutes). The increased ease of administration of tenecteplase compared to alteplase could improve interhospital transfers which is especially beneficial to countries with significant rural populations [22].

### Institutions and organizations

Australia and Canada were second and third for the countries with the most publications on tenecteplase usage in stroke. As such, it makes sense that institutions that led in publications on tenecteplase usage in stroke (the University of Melbourne, the University of Calgary, and Monash University) were located in either Australia or Canada. Each of these three institutions has had some program dedicated to understanding the efficacy of tenecteplase as a treatment for acute ischemic strokes. The University of Melbourne was the institution with the greatest number of publications on tenecteplase usage in stroke. The institution was given a grant from 2014 to 2019 to perform an analysis on Tenecteplase versus Alteplase for Stroke Thrombolysis Evaluation (TASTE). The TASTE study was an Australian-led international randomized trial designed to confirm the superiority of tenecteplase over alteplase as a clot-dissolving agent. The University of Calgary has a robust stroke research program responsible for running the largest stroke clinical trial ever run in Canada to determine the efficacy of tenecteplase for stroke treatment [23]. Figure 4 indicates that the majority of the University of Calgary’s publications on tenecteplase usage in stroke occurred in 2022. This is corroborated by the fact that the University of Calgary performed its study on the efficacy of tenecteplase as a stroke treatment in June 2022 [23].

### Yearly data

There were relatively fewer publications on tenecteplase usage in stroke between 1999 and 2010, likely due to the drug’s recent introduction to the market, which led to physicians and institutions being less familiar with the drug’s full capabilities. There was also a drop in publications on tenecteplase usage in stroke in 2020, likely due to a shift in research priorities by various institutions due to the rising importance of COVID-19. A study found that the usage of thrombolytics in various clinical studies in Asia increased after 2015 compared to before 2015 (). The increased usage of thrombolytic therapy in Asia could explain the increase in publications on tenecteplase usage in stroke after 2012 as countries around the world began increasingly recognizing the utility of thrombolytic therapy in stroke treatment. Furthermore, a National Insitute of Health (NIH) report indicated that funding for stroke research through grants, contracts, and other funding mechanisms used across the NIH increased from 308 million dollars in 2016 to 443 million dollars in 2023 [24]. 2023 was the year with the greatest funding for stroke research in the United States. The steady increase in funding for stroke research could also attempt to explain why the publications on tenecteplase usage in stroke increased throughout the years.

### Limitations

The study relied heavily on the usage of only the Web of Science to provide data points, and as such, any study not included in the Web of Science was not included in our analysis. Additionally, a previous study suggested that Web of Science may not update as quickly as other databases such as Pubmed, indicating that Web of Science may not be able to fully catalog all studies recently published [25]. Furthermore, other databases such as Scopus offer about 20% more coverage than the Web of Science [12]. As such, future studies performing bibliometric analysis should consider compiling and cross-referencing publications from multiple databases such as Web of Science, Pubmed, and Scopus to ensure the greatest accuracy in their data.

## Conclusion

Stroke is a leading cause of death and disability worldwide. As life expectancy increases, particularly in the Western world, the risk of stroke due to advanced age is greater. As such, greater research to uncover more effective treatments to reduce morbidity and mortality is warranted. In recent years, it has been stipulated that tenecteplase is non-inferior, more cost-effective, and easier to administer compared to its predecessor alteplase. Although these findings are promising, tenecteplase is still considered an off-label treatment for stroke in the US because it has not received FDA approval for treatment for acute ischemic stroke. Genentech, the drug’s manufacturer, has not yet sought federal approval for tenecteplase use in treating acute ischemic stroke.

## Data Availability

No datasets were generated or analysed during the current study.
